# Sperm cryopreservation during the SARS-CoV-2 pandemic

**DOI:** 10.1007/s40618-020-01438-8

**Published:** 2020-10-10

**Authors:** D. Paoli, F. Pallotti, G. Nigro, A. Aureli, A. Perlorca, L. Mazzuti, D. Di Carlo, O. Turriziani, A. Lenzi, F. Lombardo

**Affiliations:** 1 Laboratory of Seminology - Sperm Bank “Loredana Gandini”, Department of Experimental Medicine, “Sapienza” University of Rome, Viale del Policlinico 155, 00161 Rome, Italy; 2 Laboratory of Virology, Department of Molecular Medicine, “Sapienza” University of Rome, Rome, Italy

**Keywords:** SARS-CoV-2, COVID-19, Semen cryopreservation, Semen analysis

## Abstract

**Purpose:**

Sperm cryopreservation is fundamental in the management of patients undergoing gonadotoxic treatments. Concerns have risen in relation to SARS-CoV-2 and its potential for testicular involvement, since SARS-CoV-2-positive cryopreserved samples may have unknown effects on fertilization and embryo safety. This study therefore aimed to analyze the safety of sperm cryopreservation for cancer patients after the onset of the pandemic in Italy, through assessment of the risk of SARS-CoV-2 exposure and viral RNA testing of semen samples.

**Methods:**

We recruited 10 cancer patients (mean age 30.5 ± 9.6 years) referred to our Sperm Bank during the Italian lockdown (from March 11th to May 4th 2020) who had not undergone a nasopharyngeal swab for SARS-CoV-2 testing. Patients were administered a questionnaire on their exposure to COVID-19, and semen samples were taken. Before cryopreservation, SARS-CoV-2 RNA was extracted from a 150 µl aliquot of seminal fluid *in toto* using QIAamp viral RNA kit (Qiagen) and amplified by a real time RT PCR system (RealStar SARS-CoV2 RT PCR, Altona Diagnostics) targeting the E and S genes.

**Results:**

The questionnaire and medical interview revealed that all patients were asymptomatic and had had no previous contact with COVID-19 infected patients. All semen samples were negative for SARS-CoV-2 RNA.

**Conclusion:**

This preliminary assessment suggests that a thorough evaluation (especially in the setting of a multidisciplinary team) and molecular confirmation of the absence of SARS-CoV-2 in seminal fluid from asymptomatic cancer patients may assist in ensuring the safety of sperm cryopreservation.

## Introduction

Sperm cryopreservation has become a mainstay in the management of cancer patients undergoing genotoxic treatments capable of inducing transient or permanent sterility [1, 2]. The harmful cytological and molecular effects of cancer treatments on male gametes have been extensively studied: the high cell renewal rate of the seminiferous epithelium makes it extremely vulnerable to iatrogenic damage [3–5]. Cryopreservation in liquid nitrogen at − 196 °C can keep sperm alive indefinitely, thus enabling male fertility to be preserved. In fact, in these conditions no chemical reaction can take place, as there is insufficient thermal energy. However, literature studies have reported that viruses stored in liquid nitrogen may maintain their pathogenic properties [6]. This means that sperm cryopreservation might also preserve any viral species contaminating the semen sample. The swift spread of SARS-CoV-2, and the uncertainty caused by the paucity of data on female and male fertility in patients with COVID-19, has pushed assisted reproduction centers to seek common guidelines for both assisted reproduction techniques and gamete cryopreservation. SARS-CoV-2 shows a marked tropism for respiratory tissues, targeting types I and II pneumocytes and alveolar macrophages [7]. However, extra-respiratory transmission routes cannot be excluded. SARS-CoV-2 seems capable of interacting with angiotensin 2 converting enzyme (ACE2) [8, 9]. This is thought to enable cell invasion, and the diffuse expression of the virus in various human tissues, including the testis [10], leads to serious concerns regarding the safety of cryopreserved gametes. An exhaustive risk analysis for cryopreserved gametes and the possible consequences for embryo development is currently impossible, given the almost total lack of knowledge in this area [11]. It might therefore be important to identify SARS-CoV-2-positive patients before cryopreservation procedures for the duration of the pandemic [12], given that SARS-CoV-2 may now be present in semen samples and in liquid nitrogen in sperm banks across the world [13]. In Italy, the current recommendation is to continue performing sperm cryopreservation in cancer patients, except for those with symptoms consistent with a severe acute respiratory infection. Since nasopharyngeal swab testing was not widespread in the first stage of the pandemic and testing was limited mainly to patients with COVID-19 symptoms, it is essential to establish the suitable management of asymptomatic subjects who need to cryopreserve their semen. This study thus aimed to evaluate the safety of sperm cryopreservation of cancer patients referred to our sperm bank after the onset of the pandemic in Italy through the assessment of the risk of SARS-CoV-2 exposure and, in selected volunteers, viral RNA testing of semen samples.

## Materials and methods

### Patients

The study was approved by our University Hospital’s institutional review board (Policlinico Umberto I—“Sapienza” University of Rome Ethics Committee), and all patients gave their written informed consent. We recruited ten patients referred to the Laboratory of Seminology – Sperm Bank “*Loredana Gandini*” during the Italian lockdown (from March 11th to May 4th 2020) who had not previously undergone nasopharyngeal SARS-CoV-2 testing. SARS-CoV-2 RNA analysis was carried out in suitable semen samples before cryopreservation: patients with a semen sample volume below 2.0 ml, and patients who did not cryopreserve their semen due to azoospermia, were excluded. Information was collected on cancer type and medical history (previous diseases, medication and drug use, smoking habits and andrological diseases). Patients were also asked if they had been tested for COVID-19 and/or had had any symptoms in the previous 2 weeks.

### Semen analysis and sperm cryopreservation

Semen samples were collected by masturbation, if possible after 3–5 days of abstinence, but when treatment needed to be started urgently, sperm was stored regardless of the recorded abstinence interval. All samples were allowed to liquefy at 37 °C for 30–60 min and were then assessed according to World Health Organization Laboratory Manual [14]. The variables considered were: ejaculate volume (ml), sperm concentration (N × 10^6^/ml), total sperm number (N × 10^6^ per ejaculate), progressive motility (%), and morphology (% abnormal forms). Staff were provided with adequate personal protective equipment and samples were cryopreserved in high security straws, as normal.

### Viral RNA evaluation

SARS-CoV-2 RNA analysis was performed on 150 µl of seminal fluid *in toto*, aliquoted before the samples were processed for cryopreservation. Viral RNA was extracted using QIAamp viral RNA kit (Qiagen) according to the manufacturer’s instructions. Ten µl of extracted RNA was reverse-transcribed and simultaneously amplified using a real time RT PCR system (RealStar SARS-CoV-2 RT PCR, Altona Diagnostics) targeting the E and S genes.

### COVID-19 epidemiological questionnaire

A triage questionnaire regarding general health and respiratory symptoms was administered twice to the patients by a medical assessor: first by phone, when the patient requested an appointment, and a second time on the day of sperm cryopreservation (generally 24–48 h later).

The questionnaire investigated:

- Presence and nature of any flu-like and/or acute respiratory disease symptoms (chills, temperature > 37.5 °C, dyspnea, etc.)
- History of recent travel to or stay in countries/areas, including Italian regions and towns, with a confirmed presence of SARS-CoV-2
- Close contact with a probable or confirmed case of SARS-CoV-2 infection
- Recent admittance to a healthcare facility with confirmed cases of SARS-CoV-2.

### Statistical analysis

Data from the patients’ medical history and analyses are described as counts or percentages for categorical variables and means ± standard deviations for continuous variables.

All computations were carried out with Statistical Package for the Social Sciences (SPSS) 25.0 (SPSS Inc., Chicago, USA).

## Results

The patients’ mean age was 30.5 ± 6.9 years. The most frequently reported malignancies were hematological in nature (4 Hodgkin lymphomas, 1 mediastinal gray zone lymphoma), followed by testicular cancers (four patients) and Ewing Sarcoma (one patient). A complete caseload description is available in Table 1.

**Table 1:** Caseload description and sperm parameters (mean ± standard deviation, range in brackets)

Study group (10 subjects)	
Age at diagnosis (years)	30.5 ± 6.9 (18.0–40.0)
BMI (kg/m^2^)	23.1 ± 2.9 (18.6–29.0)
Smokers	3/10 (30%)
Cigarettes/day^a^	7.0 ± 2.6 (5–10)
Years of smoking^a^	10.0 ± 5.0 (5–15)
Abstinence (days)	6.4 ± 6.4 (2.0–10.0)
Volume (ml)	2.9 ± 1.3 (2.0–5.5)
Total sperm number (× 10^6^/ejac.)	235.7 ± 245.4 (25.6–810.0)
Progressive motility (%)	45.8 ± 14.7 (35.0–50.0)
Abnormal forms (%)	87.5 ± 4.8 (84.0–98.0)
Viability (%)	71.8 ± 18.0 (38.0–90.0)
Leukocytes (× 10^6^/ml)	1.0 ± 0.7 (0.7–1.4)
Histological diagnosis	Seminoma 4 pts (40%) Hodgkin Lymphoma 4 pts (40%) Mediastinal gray zone Lymphoma 1 pt (10%) Ewing Sarcoma 1 pts (10%)
Job	Student 3 pts (30%) Office worker 2 pts (20%) Computer programmer 1 pt (10%) Engineer 1 pt (10%) Factory worker 1 pt (10%) Sanitation worker 1 pt (10%) Unemployed 1 pt (10%)

^a^smokers only

*Semen testing for SARS-CoV-2*—We asked our patients to provide aliquots from their semen samples for SARS-CoV-2 RNA testing. The RT-PCR showed no amplifications for the E and S genes in any samples, indicating the absence of SARS-CoV-2.

*Clinical evaluation and COVID-19 questionnaire—*According to our medical interview and the answers to the COVID-19 questionnaire, no patients had fever > 37.5 °C or dyspnea on admittance to the Sperm Bank. In the previous 2 weeks all patients had been asymptomatic and had not recently traveled to or stayed in areas with a confirmed presence of SARS-CoV-2 spread, nor had any close contacts with confirmed COVID-19 cases or people with acute respiratory infections. One patient had been hospitalized, but in a ward with no confirmed cases of COVID-19 (Table 2).

**Table 2:** Medical history and COVID-19 questionnaire and testing results

Patient	Age	BMI	Pathology	Stage	Andrological history	SARS-CoV-2 naso-pharyngeal swab	COVID-19 questionnaire	SARS-CoV-2 Semen testing	Hospitalization
Patient 1	21	18.6	Ewing Sarcoma	Stage Ib	Negative	Not done	Negative	Negative	No
Patient 2	18	20.8	Hodgkin Lymphoma	Stage II	Negative	Not done	Negative	Negative	No
Patient 3	40	22.4	Hodgkin Lymphoma	Stage III	Negative	Not done	Negative	Negative	No
Patient 4	33	21.6	Testicular Cancer	Stage I	Left varicocele	Not done	Negative	Negative	No
Patient 5	35	23.4	Testicular Cancer	Stage I	Cryptorchidism (right)	Not done	Negative	Negative	No
Patient 6	31	23.1	Mediastinal Gray Zone Lymphoma	Stage III E	Negative	Not done	Negative	Negative	Yes
Patient 7	29	29.0	Testicular Cancer	Stage I	Negative	Not done	Negative	Negative	No
Patient 8	30	23.8	Hodgkin Lymphoma	Stage III	Negative	Not done	Negative	Negative	No
Patient 9	20	19.8	Hodgkin Lymphoma	Stage II	Negative	Not done	Negative	Negative	No
Patient 10	37	26.3	Testicular Cancer	Stage I	Hypermobile testis	Not done	Negative	Negative	No

## Discussion

In December 2019, Wuhan’s hospitals started to highlight cases of a severe atypical pneumonia of unknown etiology. On January 7th 2020, researchers identified a virus (now known as SARS-CoV-2) with a high homology with a bat coronavirus [15]. The swift global spread of SARS-CoV-2, its high infectiousness and its severe clinical signs [16] forced many countries to apply harsh and restrictive containment policies in an attempt to control the pandemic. In Italy, the government declared a country-wide lockdown curbing almost all activities, including those of reproductive medicine centers. The paucity of knowledge, owing to the novelty of the virus, and the unforeseen magnitude of the pandemic, forced assisted reproduction specialists to demand clear and common guidelines in relation above all to medically assisted reproduction and gamete cryopreservation. Current restrictions in Italy have led reproductive centers to drastically limit their activities, accepting only asymptomatic patients about to undergo potentially gonadotoxic treatments. For sperm banks like ours, this means above all cancer patients who urgently need to begin radio- and/or chemotherapy [17]. However, apart from safety issues for the sperm bank personnel, this novel pandemic forced us to consider three major issues: (1) does the SARS-CoV-2 virus reach the testis and the seminal fluid? If it does, (2) are there any long-lasting consequences of viral testicular infection? (3) Would the use of infected cryopreserved semen affect the fertilization process [18, 19]? While most of these questions are still lacking a definitive answer, some data in relation to other viruses in seminal fluid has been published. A number of viruses have been isolated from the seminal fluid of infected men, including replicating Zika, Ebola and Marburg viruses [20]. The troublesome possibility is that some may even show a long-term persistence in the seminal fluid, with severe repercussions for assisted reproductive technologies (ART): for example, the Zika virus has been detected in semen from asymptomatic men for up to 1 year after their recovery [21]. However, there is still little data on SARS-CoV-2. A recent paper from Li et al. reported the presence of viral RNA in the semen of around 19% of acute (four patients) and recovering (two patients) COVID-19 patients [22]. Although the limited caseload and undisclosed methods hinder us from generalizing from their results [23], the implications for reproductive medicine are alarming.

Conversely, we recently found that a symptomatic SARS-CoV-2 positive patient did not harbor viral RNA in his semen and urine during the remission phase (about a week after the positive nasopharyngeal swab) [24], and similar results were observed by Song et al. in several recovering patients [25]. Pan et al. confirmed, in a longer follow-up of 34 patients, that viral RNA was undetectable in their seminal fluid about one month after COVID-19 diagnosis [26].

The most evident difference between these studies lies in the patients recruited. Li et al. reported a caseload of patients presumably suffering from a more severe disease than those in the previous reports. Testing for the presence of SARS-CoV-2 in different stages of the disease might justify these different results. In more severe COVID-19 cases, a higher systemic diffusion of the disease might enable testicular involvement. Alternatively, in mild to moderate cases the SARS-CoV-2 clearance rate from seminal fluid might coincide with the clinical recovery. Overall, however, the evidence is still too scarce to be conclusive, and to date no evidence on sexual transmission is available.

In any case, how might SARS-CoV-2 reach the testis? We know that viruses often spread to the male reproductive tract from the blood, as the blood-testis barrier does not seem to constitute an insurmountable obstacle to viruses, especially in the presence of systemic or local inflammation [20]. To date, few studies have investigated the presence of SARS-CoV-2 in blood. Wang et al. found a minimal percentage of positive blood samples through RT-PCR amplification of viral RNA [27], whereas Zhang et al. detected the virus in 40% of blood samples [28]. It could be possible that the virus only spreads to the blood under certain circumstances, such as the acute phase or severe disease, and then to other organs such as the testis. The presence of ACE2 and TMPRSS2 may make the testis and male genital tract a viable site for the virus, but the cellular type that might function as reservoir has yet to be determined. It is therefore imperative to establish whether, when and how SARS-CoV-2 reaches the seminal fluid and how long it persists there, to assess the risks and establish strict procedures [17] for the use of gametes in assisted reproduction (transmission of infection, infection of embryos, congenital diseases, spontaneous abortions, etc.). Solving this uncertainty is even more important when we consider cryopreserved semen samples. As noted above, in this period sperm banks are mainly collecting and storing germ cells from male cancer patients. These patients have suffered persistent spermatogenetic damage from various cancer treatments and assisted reproduction may be their only chance to achieve fatherhood [3, 5, 29]. While many concerns have been raised in relation to the collection, shipping and use of these samples [13], sperm banks in Italy are currently permitting sperm cryopreservation for asymptomatic subjects, in accordance with local indications.

From the little data available, the risk that SARS-CoV-2 might spread to the testis and be transmitted through the seminal fluid seems fairly low in mild to moderate COVID-19 patients and, although no investigation has been conducted in asymptomatic subjects, the chance of SARS-CoV-2 being present in our asymptomatic cancer patients should be even lower. However, the prevalence of asymptomatic subjects and their contribution to the transmission of COVID-19 is not well characterized [30], and their role in the spread of the virus poses a tremendous epidemiological challenge [31]. These subjects must be identified, and a careful clinical evaluation and contact history may still be the only way to do so [31].

For this reason, the patients at our sperm bank underwent a detailed medical history and epidemiological questionnaire to minimize the risk. This was further confirmed by testing seminal fluid samples from 10 asymptomatic cancer patients for SARS-CoV-2 RNA. As mentioned above, no viral RNA was found in any sample, suggesting that asymptomatic cancer patients with no previously known SARS-CoV-2 infection have a very low chance of harboring this coronavirus in their semen.

In our experience, SARS-CoV-2 testing of patients referred for sperm banking, while recommended [12], was inadequate at the onset of the pandemic. While a screening strategy with nasopharyngeal swabs or serological testing is advisable in all cancer patients, the large numbers of symptomatic inpatients at the start of the pandemic made this unfeasible. In the absence of SARS-CoV-2 testing, it is important to distinguish between asymptomatic patients who have, and those who have not, been at risk of exposure, to select patients whose cryopreserved gametes present the smallest residual risk of harboring the virus. The inclusion of this test in cryopreservation protocols during the pandemic could further ensure the safety of sperm cryopreservation performed in this period.

From this perspective, the absence of viral RNA in our cryopreserved samples has a double value. First, it comprises a preliminary biomonitoring for SARS CoV-2 in the semen of asymptomatic cancer patients. Second, it underlines the importance of taking a thorough medical history, accompanied in our case by the administration of an epidemiological questionnaire. This careful evaluation (especially in the setting of a multidisciplinary team, where the ART specialist works in tandem with the oncologist and infectious disease specialist/virologist), together with the molecular confirmation of the virus’ absence in the semen sample, should help ensure the safety of sperm cryopreservation.

This precautionary approach, together with suitable personal protective equipment and the use of high security straws, should minimize the risks associated with cryopreserving sperm during the pandemic. Furthermore, the current evidence seems increasingly to point to the absence of SARS CoV-2 in semen. The risk of preserving contaminated semen samples is thus minimal, provided that all necessary precautions and safety procedures are observed. Nonetheless, it should be stressed that these results are not conclusive, as they refer to a very small caseload, and need further confirmation, especially as we focused on the safety issues associated with possible asymptomatic COVID-19 infections, without investigating whether SARS-CoV-2 infection is capable of spreading to the testis and seminal fluid.
